# Usefulness of MALDI-TOF MS as a Diagnostic Tool for the Identification of *Streptococcus* Species Recovered from Clinical Specimens of Pigs

**DOI:** 10.1371/journal.pone.0170784

**Published:** 2017-01-26

**Authors:** Marta Pérez-Sancho, Ana I. Vela, Teresa García-Seco, Sergio González, Lucas Domínguez, Jose Francisco Fernández-Garayzábal

**Affiliations:** 1 Centro de Vigilancia Sanitaria Veterinaria (VISAVET), Universidad Complutense, Madrid, Spain; 2 Campus de Excelencia Internacional (CEI) Moncloa, Universidad Politécnica de Madrid (UPM)-Universidad Complutense de Madrid (UCM), Madrid, Spain; 3 Departamento de Sanidad Animal, Facultad de Veterinaria, Universidad Complutense, Madrid, Spain; Cornell University, UNITED STATES

## Abstract

The application of MALDI-TOF MS for identifying streptococcal isolates recovered from clinical specimens of diseased pigs was evaluated. For this proposal, the MALDI BDAL Database (Bruker Daltoniks, Germany) was supplemented with the main spectrum profiles (MSP) of the reference strains of *S*. *porci*, *S*. *porcorum* and *S*. *plurextorum* associated with pneumonia and septicemia. Although these three species showed similar MALDI profiles, several peaks were recognized that can be useful for their differentiation: *S*. *porci* (4113, 6133, 7975 and 8228 m/z Da), *S*. *plurextorum* (3979, 4078, 4665, 6164, 6491, 6812, 7959 and 9330 m/z Da) and *S*. *porcorum* (3385, 3954, 4190, 6772, 7908, and 8381 m/z Da). After adding these MSPs, an evaluation was conducted to determine the accuracy of MALDI-TOF MS for the identification of streptococci from diseased pigs using 74 field isolates. Isolates were identified as *S*. *suis*, *S*. *porcinus*, *S*. *dysgalactiae*, *S*. *hyovaginalis*, *S*. *porcorum*, *S*. *alactolyticus*, *S*. *hyointestinalis* and *S*. *orisratti*. This is the first time that the latter three species have been reported from clinical specimens of pigs. Overall, there was good concordance (95.9%) between the results obtained from MALDI-TOF MS identification (best hint) and those from genotyping. Our results demonstrate the good performance of MALDI-TOF MS (100% sensitivity and specificity) for identifying most of the species of streptococci that can frequently be isolated from diseased pigs. However, conflicting results were observed in the correct identification of some isolates of *S*. *dysgalactiae* and *S*. *alactolyticus*.

## Introduction

Several species of streptococci, such as *Streptococcus suis*, *Streptococcus dysgalactiae* subsp. *equisimilis* and *Streptococcus porcinus* are well-recognized swine pathogens [1, 2]. *S*. *suis* is by far the most important agent of infectious diseases in this group. It is associated with a variety of clinical conditions including meningitis, septicemia, arthritis, endocarditis and pneumonia. It has also been isolated from cases of rhinitis and abortion. *S*. *dysgalactiae* subsp. *equisimilis* causes sporadic cases of septicemia and arthritis in sucking pigs, endocarditis in growing pigs and ascending infection of the uterus in sows [1, 3]. *S*. *porcinus* is the etiological agent of streptococcal lymphadenitis in growing pigs. It also causes throat abscesses and has sometimes been isolated from pneumonia [1, 4, 5]. Other *Streptococcus* species have been isolated from clinical specimens in swine. *Streptococcus plurextorum* has been isolated from lesions associated with pneumonia and septicemia [6] and *Streptococcus porci* and *Streptococcus porcorum* have also been associated with pneumonia in pigs [7, 8]. However, there are no data on the prevalence and clinical significance of these three latter species.

Routine identification and differentiation of the *Streptococcus* species is traditionally based on phenotypic/biochemical techniques. However, identifying the *Streptococcus* species using these tests can be difficult [9, 10]. The application of matrix-assisted laser desorption/ionization time-of-flight mass spectrometry (MALDI-TOF MS) has greatly facilitated microbial bacterial identification in clinical microbiology laboratories, mainly because it provides improved accuracy and power of resolution for identifying microbial isolates, in addition to a decreasing turn-around time in comparison to conventional methods [11]. This technique has been widely used for the identification of many different bacteria, including streptococci [12–14]. In particular, MALDI-TOF MS has proved to be an excellent and reliable alternative to PCR-based methods for routine identification of *S*. *suis* [15]. However, *S*. *plurextorum*, *S*. *porci* and *S*. *porcorum* are species that are phylogenetically closely related to *S*. *suis* but they have not been evaluated yet by MALDI-TOF MS. Therefore, the aim of the present work was to incorporate the main spectrum profiles (MSP) of reference strains of *S*. *porci*, *S*. *porcorum* and *S*. *plurextorum* into the MALDI BDAL Database (5627 entries; Bruker Daltoniks, Germany) and then explore the usefulness of the MALDI-TOF MS technology as a diagnostic tool for the identification of streptococci associated with infections in pigs.

## Materials and Methods

The present work does not include any experimental infection trials with pigs. Only diseased farmed pigs were used to microbiologically identify the etiological agent of the disease. We did not, therefore, consult with the Institutional Animal Care and Use Committee and no specific national regulations for these procedures are available. Diseased pigs were necropsied under strict hygienic conditions by the veterinarian in the farms immediately after the pig´s death and clinical specimens were sent to the Health Surveillance VISAVET Centre of the Universidad Complutense (Madrid, Spain) for a confirmatory microbiological diagnosis. Clinical specimens were handled following the recommendations of the OIE for the transport of specimens of animal origin (http://asforce.org/course/assets/img/module2/transport.pdf). Upon arrival, clinical samples were processed in biological safety cabinets within 12h. Samples were cultured on Columbia and Columbia-CNA agar plates that were incubated at 37°C for 24h under aerobic conditions.

### *Streptococcus* strains

A first set of strains was used to create the Main Spectrum Profiles (MSP) to be included in the Bruker MALDI BDAL database (version 3.4; 5627 entries): *S*. *plurextorum* (1956-02^T^ and 1355–03), *S*. *porci* (2923-03^T^ and 2857–03), and *S*. *porcorum* (682-03^T^ and 1792–03). These strains were identified during polyphasic taxonomic studies that guaranteed their correct identification. These taxonomic studies included biochemical, genetic and phylogenetic analysis of the isolates [6–8].

After the MSPs of these species were defined and incorporated into the MALDI- BDAL Database (Bruker Daltoniks, Germany), a second set of 74 additional field isolates was used to validate the utility of the MALDI-TOF MS approach for the diagnosis of diseases in pigs caused by these bacteria. Isolates were originated from clinical samples of diseased pigs (S1 Table). MALDI-TOF MS identification was confirmed in the isolates using different genetic approaches that included sequencing of the 16S rRNA gene or species-specific PCR assays. For 16S rRNA gene sequence analysis a large continuous fragment (approximately 1400 bases) of the 16S rRNA gene of the remaining isolates was determined from PCR-amplified products, derived from universal primers pA (5′-agagtttgatcctggctcag-3′) and pH* (5′-aaggaggtgatccagccgca-3′) and the amplified product was further sequenced bidirectionally [16]. The 16S rRNA gene sequences of isolates were further compared with those available on the EzTaxon, server (http://eztaxon-e.ezbiocloud.net/). All the strains were grown on Columbia sheep agar plates at 37°C under aerobic conditions for 24h and single colonies were selected for MALDI-TOF MS analysis. For *S*. *suis* isolates, identification was determined by a *S*. *suis* species-specific PCR [17] that amplified a 688-bp fragment of the glutamate dehydrogenase gene (*gdh*).

### Preparation of *Streptococcus* spp. cell lysates and conditions for MALDI-TOF MS profile determination

Cells from representative bacterial colonies were re-suspended in 300μL of HPLC-grade water and mixed vigorously prior to 900μL of HPLC-grade ethanol being added. Subsequently, an acetonitrile/formic acid extraction protocol was performed in accordance with the manufacturer’s instructions (Bruker Daltonik, Bremen, Germany). After protein extraction, one microliter of each isolate extract was spotted onto a 384-spot polished steel target plate, let to dry at room temperature and overlaid with one microliter of α-cyano-4-hydroxy-cinnamic acid (HCCA) matrix. Data acquisition was conducted in linear positive ion mode using a Bruker Daltonik UltrafleXtreme MALDI TOF/TOF system with a set mass range of 2-20KDa and a nitrogen laser (335 nm) for ionization of the molecules. The specific settings for spectra acquisition were: laser frequency: 2000 Hz, acceleration voltage: 25 kV. Each spectrum was acquired using FlexControl software (Version 3.4) in automatic mode in a random sampling pattern.

### Proteomic approach for the identification and classification of *Streptococcus* isolates

Six new entries (*S*. *plurextorum*, n = 2; *S*. *porci*, n = 2 and *S*. *porcorum*, n = 2) were entered manually in our MALDI database, which included all the Bruker MALDI BDAL database entries plus three entries of *S*. *suis* serotypes 2, 7 and 9 as described previously [15]. To create the MSPs of *S*. *plurextorum*, *S*. *porci* and *S*. *porcorum*, a total of 24 spectra per isolate were obtained from the 8 spots in three measurements. Spectra were analyzed by FlexAnalysis (version 3.0, Bruker Daltonics) according to [18]. Briefly, after smoothing, normalization, baseline subtraction and peak picking, a minimum of 20 high-quality spectra per strain were selected for MSP creation. High-quality spectra for each species were downloaded using MALDI Biotyper OffLine Classification Software (version 3.0, Bruker Daltonics) to create a main spectrum profile (MSP) for each strain with default settings in the automatic mode. Finally, the presence and masses of species-identifying biomarker ions for *S*. *porcorum*, *S*. *porci* and *S*. *plurextorum* were evaluated using FlexAnalysis software.

After inclusion of the new MSPs, a panel of 74 *Streptococcus* isolates recovered from clinical specimens of swine were identified using the MALDI Biotyper Offline Classification software (version 3.1). The reliability of the identification was evaluated according to the log (score) values, calculated with the MALDI Biotyper software mentioned above. Interpretation of the score value obtained for each isolate was performed according to the manufacturer’s instructions: values between 3.000–2.300 indicated highly probable species identification, values from 2.299 to 2.000 indicated secure genus and probable species identification, while lower scores between 1.999 and 1.700 were considered as probable genus identification. The Biotyper software showed a total of 10 top matches (defined by the user) in an identification ranking list ordered according to the score value of each identification.

### Data analysis

The identification accuracy of MALDI-TOF MS (sensitivity, specificity and 95% confidence intervals) for each streptococcal species under study was estimated using WINPEPI (PEPI-for-Windows) v. 11.35 [19].

## Results

**Fig 1:**
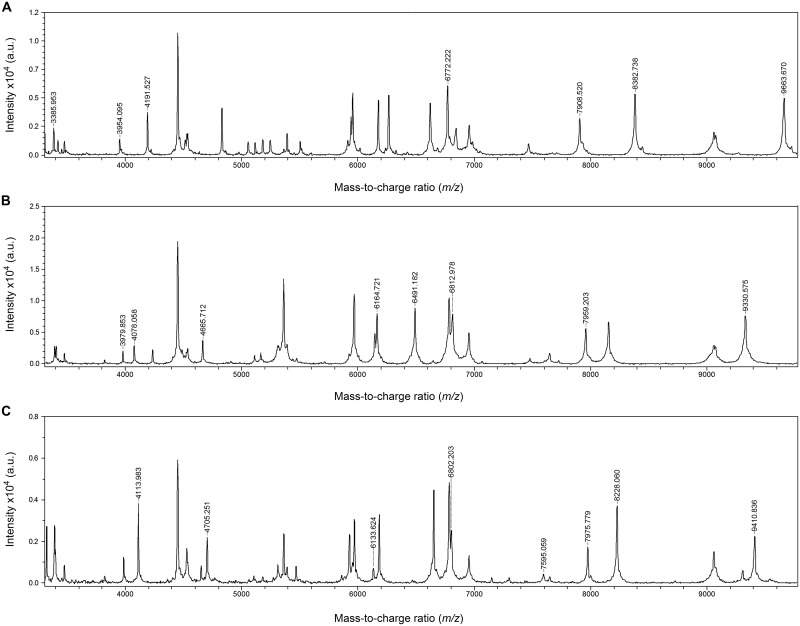
MALDI-TOF MS spectra of whole-cell extracts of *S*. *porcorum* (A), *S*. *plurextorum* (B) and *S*. *porci* (C) reference strains. The relative intensities of the ions are shown on the *y* axis, and the masses (in Da) of the ions are shown on the *x* axis.

Analysis of the spectra of these strains was performed by selecting a range of m/z between 4000 and 9500 Da that included the major differences between their MSPs. The spectra of *S*. *porci* and *S*. *plurextorum* were quite similar, but closer analysis of their mass peak profiles revealed some different mass patterns for each species (Fig 1).

Both strains of *S*. *porci* showed the presence of indicative peaks at 4113, 6133, 7975 and 8228 m/z Da. Both strains of *S*. *plurextorum* presented representative peaks at 3979, 4078, 4665, 6164, 6491, 6812, 7959 and 9330 m/z Da. Similarly, the spectra of both strains of *S*. *porcorum* showed representative peaks at 3385, 3954, 4190, 6772, 7908, and 8381 m/z Da. These data indicate the existence of important differences between the spectra of these three species. Spectra of these three species were further compared to the spectra of the clinical pig-associated *Streptococcus* species. Table 1 shows the indicative species-specific m/z peaks for each of these species that may be useful for their differentiation.

**Table 1:** MALDI-TOF MS indicative peak masses of the different streptococcal species isolated from clinical specimens in pigs.

	Peak Masses Values (m/z)	Intensity (a.u)	Signal to Noise(s/n)	Frequency (total test isolates)
*S*. *porcorum*	4190	1965–10654	14–48	100% (6)
	8381	3293–19350	25–100	100% (6)
*S*. *plurextorum*	6164	2660–13354	28–59	100% (2)
*S*. *porci*	6133	635–12146	9–56	100% (2)
*S*. *suis*	4133	836–21988	9–71	100% (36)
	8267	1433–19065	30–115	100% (36)
*S porcinus*^a^	6354	6330–13414	49–81	100% (8)
	9456	7070–20009	87–193	100% (8)
*S*. *dysgalactiae*	8182	2317–15252	23–82	100% (8)
	9486	832–16557	8–75	100% (8)

^a^ Four *S*. *suis* isolates showed peaks of 6357–6358 Da with s/n value of 4–5 and relative intensities of 774–1317 a.u. and one isolate showed the peak of 9458 Da with s/n and a.u. values of 6 and 795, respectively.

**Fig 2:**
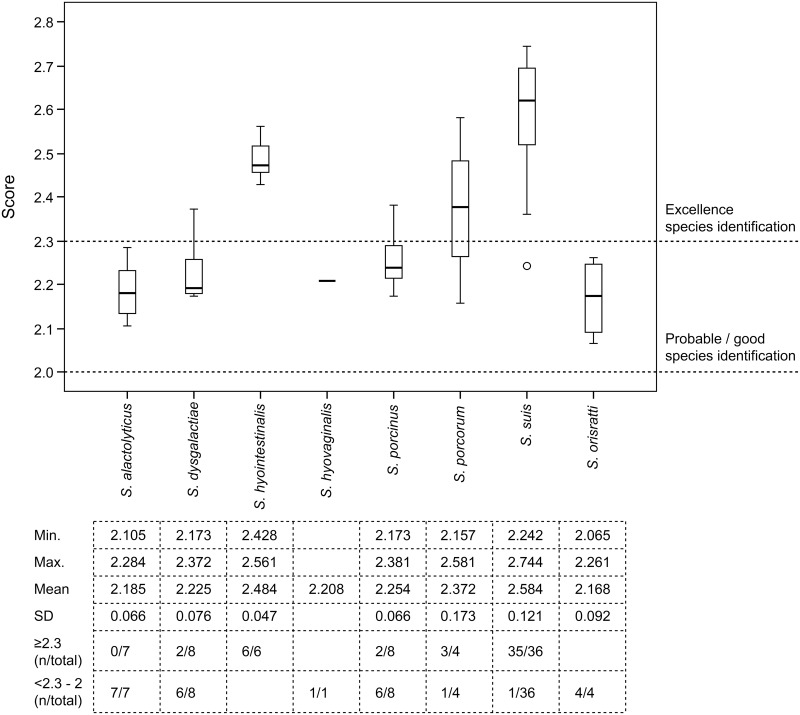
Box-and-whisker plots of the distribution of mean score values of *Streptococcus* species potentially isolated from clinical specimens of swine. Two cut-off values were established for excellent (≥2.300) and good (2.000–2.299) identification at species level.

Before the MSPs of *S*. *porci*, *S*. *porcorum* and *S*. *plurextorum* were incorporated into the Bruker database (version 3.4; 5627 entries) the isolates of these species gave identification score values lower than 1.900, which indicates these species would be clearly differentiated from any of the *Streptococcus* species currently included in the mentioned database, including those species associated with disease in pigs. After the MSPs of these streptococcal species were incorporated into the Bruker MALDI BDAL database, the utility of MALDI-TOF MS for identifying those species of streptococci associated with disease in pigs was evaluated with 74 streptococcal field isolates recovered from different clinical specimens of diseased pigs (S1 Table). The results of genetic and MALDI-TOF MS-based identification of these isolates (first option in the identification ranking list) are shown in Table 2 and the average and range of identification score values obtained for each *Streptococcus* species are shown in Fig 2. Further information of each test *Streptococcus* isolate is included as S1 Table.

**Table 2:** Results of MALDI-TOF MS and genotype identification of the 74 streptococcal clinical isolates from diseased pigs examined in this study.

Genotypic Identification^a^	N° isolates			Score average (range)	MALDI-TOF MSIdentification^b^	Lesions
	Total	>2.3	2.299–2.0			
*S*. *suis*	36	35	1	2.584 (2.744–2.242)	*S*. *suis*	Septicemia, pneumonia, arthritis, endocarditis, meningitis
*S*. *hyointestinales*	6	6	-	2.484 (2.561–2.428)	*S*. *hyointestinales*	Pneumonia, endocarditis
*S*. *porcinus*	8	2	6	2.254 (2.381–2.173)	*S*. *porcinus*	Pneumonia, arthritis, endocarditis
*S*. *dysgalactiae*	8	2	6	2.225 (2.372–2.173)	*S*. *dysgalactiae*^c^	Pneumonia, arthritis, endocarditis
*S*. *alactolyticus*	7	-	7	2.213 (2.284–2.145) 2.149 (2.220–2.105)	*S*. *alactolyticus* (n = 4)^d^ *S*. *lutetiensis* (n = 3)	Pneumonia, arthritis
*S*. *orisratti*	4	-	4	2.168 (2.261–2.065)	*S*. *orisratti*	Pneumonia, endocarditis
*S*. *hyovaginalis*	1	-	1	2.208	*S*. *hyovaginalis*	Arthritis
*S*. *porcorum*	4	3	1	2.372 (2.157–2.581)	*S*. *porcorum*	Pneumonia

^a^ All isolates were genotypically identified by different genetic approaches that included sequencing of their 16S rRNA gene or species-specific PCR assays.

^b^ Results of the first option in the identification ranking list provided by the MALDI Biotyper. Except for *S*. *dysgalactiae* and *S*. *alactolyticus*, the second identification option in all isolates of the other streptococcal species gave always score values of <2.0.

^c^ In 5 isolates, the second identification options were *S*. *equi*, *S*. *canis* or *S*. *pyogenes*, with score values of >2.000.

^d^ The second identification options of these isolates were *S*. *lutetiensis*, *S*. *equinus* or *S*. *gallolyticus* also with score values of >2.000.

The results of MALDI-TOF MS identification (best hint) were concordant in most isolates with that of genetic identification (95.9%, 71/74). More than half of the isolates (48/74, 64.8%) showed score values higher than 2.300, while the remaining isolates (26/74, 35.1%) presented scores between 2.299–2.000, values considered as probable identification at species level (Fig 2). Evaluation of the accuracy of MALDI-TOF MS was determined by calculating its sensitivity and specificity for each of the *Streptococcus* species included in this study. Except for *S*. *alactolyticus* and *S*. *dysgalactiae*, the sensitivity and specificity values were 100% for the remaining streptococcal species (95% CI: 20.7–100 and 90.8–100 depending on the species, respectively). Specificity values for *S*. *alactolyticus* and *S*. *dysgalactiae* were also 100%, but the sensitivity values were 0% (95% CI: 0–35.43) and 37.5% (95% CI: 13.68–69.43), respectively, considering erroneous identifications (Table 2) those isolates that were misidentified (3 *S*. *alactolyticu*s isolates) and those with inconsistent identifications (second identification option with score values >2.000; 4 *S*. *alactolyticus* and 5 *S*. *dysgalactiae*).

The MALDI-TOF MS system accurately identified the species *S*. *suis*, *S*. *porcinus*, *S*. *hyointestinalis*, *S*. *hyovaginalis*, *S*. *porcorum* and *S*. *orisratti* as all isolates of these species were identified with score values >2.0 while was the second identification option gave always score values of <2.0 (Table 2). Limitations of the MALDI-TOF MS system were observed for the identification of *S*. *alactolyticus* and *S*. *dysgalactiae*. In the case of *S*. *alactolyticus*, this species was identified as a first option, with score values between 2.284 and 2.145 in four isolates (Table 2), although the second most probable identification option of these isolates were *Streptococcus lutetiensis*, *Streptococcus equinus* or *Streptococcus gallolyticus*, also with score values >2.0 (Table 2). The remaining three isolates of *S*. *alactolyticus* were identified as first option as *S*. *lutetiensis*, with score values between 2.220 and 2.105 (Table 2). The eight isolates of *S*. *dysgalactiae* were always identified as this species as first option with score values between 2.372 and 2.173 (Table 2), but in 5 out of the 8 isolates the second most probable identification options were *Streptococcus equi*, *Streptococcus canis* or *Streptococcus pyogenes*, also with score values of >2.0 (Table 2).

## Discussion

Among the *Streptococcus* species associated with infections in pigs [1], only *S*. *suis*, *S*. *dysgalactiae* and *S*. *porcinus* were included in the Bruker MALDI BDAL database (Bruker Daltoniks) at the time of this study. However, the species *S*. *porci*, *S*. *porcorum* and *S*. *plurextorum* associated with pneumonia and septicemia in pigs [6–8] were not included, which limits the utility of this technology for the diagnosis of diseases caused by streptococci. Therefore, the first objective of this study was to construct the MSPs of *S*. *porci*, *S*. *porcorum* and *S*. *plurextorum* and insert them into the Bruker database. Analysis of the MSPs of these three species as well as those of *S*. *suis*, *S*. *dysgalactiae* and *S*. *porcinus* identified some species-specific m/z peaks that exhibited good discriminatory power based on the data of their intensity, signal-to-noise and frequency of detection (Table 1), and therefore likely useful for the differentiation of these six species of streptococci. The reproducibility of mass spectra profiles could be affected by intraspecies variability [20]. Therefore, future analysis of the mass spectra of more isolates of these species will allow confirmation of whether these biomarkers are consistently present in the majority of their respective species. We further evaluated the efficacy of MALDI-TOF MS for the identification of streptococci associated with infections in pigs using a panel of 74 field clinical isolates (S1 Table). Overall, there was good concordance between the results of identification obtained with the MALDI-TOF MS system and those obtained by genetic identification, with both approaches giving a matching identification for 95.9% of the streptococci considering the best hint identification of MALDI-TOF MS (Table 2). However a certain dispersion in the estimation (95% CI: 20.7–100 for sensitivity and 95% CI: 90.8–100 for specificity) could be due to the limited number of isolates in some *Streptococcus* species here evaluated. Most field isolates (70.3%) were identified as *S*. *suis*, *S*. *porcinus* or *S*. *dysgalactiae*, a result that is in line with the epidemiological relevance of these pathogens in the swine industry [1,2]. The relatively high number of clinical isolates of the species *S*. *hyointestinalis*, *S*. *alactolyticus* and *Streptococcus orisratti* was also interesting (Table 2). The two former species are considered to be part of the microflora in the intestine [1] and *S*. *orisratti* was initially isolated from the teeth of healthy rats [21]. Isolates of these species were recovered from lesions associated with pneumonia, endocarditis or arthritis (Table 2) and therefore, this result represents the first report of the isolation of these species from clinical specimens of pigs. However, no definitive conclusions about the clinical significance of these species as pig pathogens can be reached from the results of this study.

Isolates of *S*. *suis*, *S*. *hyointestinalis* and *S*. *porcorum* were correctly identified by MALDI-TOF MS with average scores of 2.584, 2.484 and 2.372, respectively (Table 2; Fig 2). Similarly *S*. *porcinus*, *S*. *hyovaginalis* and *S*. *orisratti* isolates were also correctly identified by the MALDI-TOF MS system with score values between 2.381 and 2.065 (Table 2). The sensitivity and specificity of MALDI-TOF MS for these species were 100%, indicating the good performance of MALDI-TOF MS approach as a diagnostic tool for their identification. To our knowledge, no specific studies for the identification of *S*. *hyointestinalis* or *S*. *porcorum* are available, but the results for *S*. *suis* and *S*. *porcinus* agree with the good performance of MALDI-TOF MS for identification of these species [14,15]. However, limitations of the MALDI-TOF MS system were observed for the identification of *S*. *alactolyticus* and *S*. *dysgalactiae* isolates. *S*. *alactolyticus* was not clearly discriminated from *S*. *lutetiensis*, *S*. *equinus* or *S*. *gallolyticus* (Table 2) as these species also appeared in the second best match identification list with score values of >2.0 (Table 2). All these species belong to the phylogenetically closely-related bacterial complex “bovis group” or the *Streptococcus bovis/equinus*-complex [22] and the results of this study corroborate the limitations of MALDI-TOF MS for differentiating members of the *S*. *bovis/equinus* complex [23, 24]. Similarly, the inconsistency of MALDI-TOF MS for identifying *S*. *dysgalactiae* (Table 2) has also been observed previously [24].

## Conclusions

Despite some limitations regarding the identification of *S*. *dysgalactiae* and *S*. *alactolyticus*, the results of this study confirm that MALDI-TOF MS represents a good diagnostic tool for identifying most of the species of streptococci that are frequently isolated from diseased pigs and therefore for the diagnosis of disease caused by these bacteria.

## Supporting Information

S1 Table Details of the 74 field streptococci isolates included in the study. (DOC)
