# Supplementary material for: Usefulness of MALDI-TOF MS as a Diagnostic Tool for the Identification of Streptococcus Species Recovered from Clinical Specimens of Pigs
Source: PLoS One. 2017 Jan 26;12(1):e0170784. doi: 10.1371/journal.pone.0170784 (PMC5268416; doi:10.1371/journal.pone.0170784)
Supplement: S1 Table — (DOC) [file pone.0170784.s001.doc]

Table S1. Details of the 74 field streptococci isolates included in the study.

| ***S.* species^a^** | **Isolate** | **Farm** | **Year** | **MALDI-TOF Identification^b^** | **Score value** |
| --- | --- | --- | --- | --- | --- |
| *S. alactolyticus* | 484/03a | 3 | 2003 | *S. alactolyticus* | 2.180 |
|  | C 1895/03 | 11 | 2003 | *S. alactolyticus* | 2.243 |
|  | C 1107/03A | 17 | 2003 | *S. lutetiensis* | 2.105 |
|  | C05/0011-13P1 | 25 | 2005 | *S. alactolyticus* | 2.284 |
|  | C 597/03A | 43 | 2003 | *S. alactolyticus* | 2.145 |
|  | C 3430/03 | 46 | 2003 | *S. lutetiensis* | 2.122 |
|  | C 203/04 | 46 | 2004 | *S. lutetiensis* | 2.22 |
| *S. dysgalactiae* | C04/1293-09Z1 | 20 | 2004 | *S. dysgalactiae* | 2.319 |
|  | C 661/03 | 12 | 2003 | *S. dysgalactiae* | 2.173 |
|  | C 722/03B | 28 | 2003 | *S. dysgalactiae* | 2.185 |
|  | C04/1360-04Ñ1 | 20 | 2004 | *S. dysgalactiae* | 2.190 |
|  | C 1640/03 | 6 | 2003 | *S. dysgalactiae* | 2.372 |
|  | C 2950B/03 | 19 | 2003 | *S. dysgalactiae* | 2.173 |
|  | C 1386/03A | 24 | 2003 | *S. dysgalactiae* | 2.193 |
|  | C 893C/04 | 38 | 2004 | *S. dysgalactiae* | 2.195 |
| *S. hyointestinalis* | C 1067B/03 | 22 | 2003 | *S. hyointestinalis* | 2.456 |
|  | 1110A/02 | 46 | 2002 | *S. hyointestinalis* | 2.469 |
|  | C 212B/04 | 14 | 2004 | *S. hyointestinalis* | 2.475 |
|  | C 1332B/03 | 23 | 2003 | *S. hyointestinalis* | 2.561 |
|  | 78C/02 | 40 | 2002 | *S. hyointestinalis* | 2.428 |
|  | C 3587/03 | 47 | 2003 | *S. hyointestinalis* | 2.516 |
| *S. hyovaginalis* | C04/1560-01G*1 | 36 | 2004 | *S. hyovaginalis* | 2.208 |
| *S. porcinus* | C04/1293-02T1 | 20 | 2004 | *S. porcinus* | 2.253 |
|  | C 576/03B | 28 | 2003 | *S. porcinus* | 2.231 |
|  | C 1863B/03 | 37 | 2003 | *S. porcinus* | 2.381 |
|  | C 3370B/03 | 2 | 2003 | *S. porcinus* | 2.216 |
|  | C 2603B/03 | 15 | 2003 | *S. porcinus* | 2.324 |
|  | C04/1456-02T2 | 18 | 2004 | *S. porcinus* | 2.173 |
|  | C 2667A/03 | 29 | 2003 | *S. porcinus* | 2.245 |
|  | C04/1497-09T2 | 20 | 2004 | *S. porcinus* | 2.212 |
| *S. porcorum* | C04/1561-9D2-04 | 8 | 2004 | *S. porcorum* | 2.370 |
|  | DICM08-00279-1A | 8 | 2008 | *S. porcorum* | 2.383 |
|  | 1606-02 | 45 | 2002 | *S. porcorum* | 2.157 |
|  | C04/1561-2D2 | 8 | 2004 | *S. porcorum* | 2.581 |
| *S. orisratti* | C04/1341-05Z1 | 20 | 2004 | *S. orisratti* | 2.231 |
|  | C 2051/03 | 31 | 2003 | *S. orisratti* | 2.116 |
|  | C 2155A/03 | 41 | 2003 | *S. orisratti* | 2.065 |
|  | C 1890A/03 | 44 | 2003 | *S. orisratti* | 2.261 |
| *S. suis* | 656/2 | Co-1 | 2002 | *S. suis* | 2.586 |
|  | 668/2 | Co-3 | 2002 | *S. suis* | 2.510 |
|  | 14 | Co-9 | 1999 | *S. suis* | 2.626 |
|  | 238/03 | 1 | 2003 | *S. suis* | 2.618 |
|  | 666/02 | 4 | 2003 | *S. suis* | 2.704 |
|  | 255/01 | 9 | 2001 | *S. suis* | 2.381 |
|  | 7 | 21 | 1999 | *S. suis* | 2.528 |
|  | 18 | 30 | 1999 | *S. suis* | 2.414 |
|  | 306/03 | 42 | 2003 | *S. suis* | 2.560 |
|  | 139/01 | 46 | 1999 | *S. suis* | 2.744 |
|  | 363/01 | 27 | 2001 | *S. suis* | 2.551 |
|  | 404/01 | 28 | 2001 | *S. suis* | 2.721 |
|  | C04/1432-01Z1 | 32 | 2004 | *S. suis* | 2.696 |
|  | 117/01 | 16 | 2001 | *S. suis* | 2.701 |
|  | C04/1236-03L1 | 13 | 2004 | *S. suis* | 2.662 |
|  | 556/02 | 13 | 2002 | *S. suis* | 2.480 |
|  | C04/1253-01P1 | 5 | 2004 | *S. suis* | 2.622 |
|  | C04/1208-03P | 13 | 2004 | *S. suis* | 2.535 |
|  | C 3236/03 | 26 | 2003 | *S. suis* | 2.531 |
|  | 632/01 | 34 | 2001 | *S. suis* | 2.676 |
|  | 121A/02 | 40 | 2002 | *S. suis* | 2.522 |
|  | 477/01 | 35 | 2001 | *S. suis* | 2.516 |
|  | C 2649A/03 | 39 | 2003 | *S. suis* | 2.635 |
|  | 1738 | 7 | 1999 | *S. suis* | 2.685 |
|  | C 2995A/03 | 10 | 2003 | *S. suis* | 2.480 |
|  | C 933B/04 | 14 | 2004 | *S. suis* | 2.700 |
|  | C05/0011-05T1 | 25 | 2005 | *S. suis* | 2.641 |
|  | 1746/2 | 33 | 1999 | *S. suis* | 2.710 |
|  | 517/02 | 33 | 2002 | *S. suis* | 2.650 |
|  | 798 | 48 | 1999 | *S. suis* | 2.360 |
|  | 1421/3 | 49 | 1999 | *S. suis* | 2.390 |
|  | 1428/3 | 49 | 1999 | *S. suis* | 2.561 |
|  | 1429/3 | 49 | 1999 | *S. suis* | 2.721 |
|  | 1432/3 | 49 | 1999 | *S. suis* | 2.703 |
|  | 1407/3 | 49 | 1999 | *S. suis* | 2.242 |
|  | 1410/3 | 49 | 1999 | *S. suis* | 2.692 |

^a^ Genetic identification was achieved by sequencing of the 16S rRNA gene or by species-specific PCR assays (see material and methods)

^b^ Result of the best match in the Biotyper identification list
